# The NOD2 p.Leu1007fsX1008 Mutation (rs2066847) Is a Stronger Predictor of the Clinical Course of Crohn's Disease than the *FOXO3A* Intron Variant rs12212067

**DOI:** 10.1371/journal.pone.0108503

**Published:** 2014-11-03

**Authors:** Fabian Schnitzler, Matthias Friedrich, Christiane Wolf, Marianne Angelberger, Julia Diegelmann, Torsten Olszak, Florian Beigel, Cornelia Tillack, Johannes Stallhofer, Burkhard Göke, Jürgen Glas, Peter Lohse, Stephan Brand

**Affiliations:** 1 Department of Medicine II-Grosshadern, Ludwig-Maximilians-University (LMU), Munich, Germany; 2 Department of Preventive Dentistry and Periodontology, LMU, Munich, Germany; 3 Max-Planck-Institute of Psychiatry, Munich, Germany; 4 Department of Human Genetics, Rheinisch-Westfälische Technische Hochschule (RWTH), Aachen, Germany; 5 Department of Clinical Chemistry-Grosshadern, LMU, Munich, Germany; Innsbruck Medical University, Austria

## Abstract

**Background:**

Very recently, a sub-analysis of genome-wide association scans revealed that the non-coding single nucleotide polymorphism (SNP) rs12212067 in the *FOXO3A* gene is associated with a milder course of Crohn's disease (CD) (*Cell* 2013;155:57–69). The aim of our study was to evaluate the clinical value of the SNP rs12212067 in predicting the severity of CD by correlating CD patient genotype status with the most relevant complications of CD such as stenoses, fistulas, and CD-related surgery.

**Methodology/Principal Findings:**

We genotyped 550 CD patients for rs12212067 (*FOXO3A*) and the three common CD-associated *NOD2* mutations rs2066844, rs2066847, and rs2066847 and performed genotype-phenotype analyses.

**Results:**

No significant phenotypic differences were found between the wild-type genotype TT of the *FOXO3A* SNP rs12212067 and the minor genotypes TG and GG independently from *NOD2* variants. The allele frequency of the minor G allele was 12.7%. Age at diagnosis, disease duration, body mass index, surgery rate, stenoses, fistula, need for immunosuppressive therapy, and disease course were not significantly different. In contrast, the NOD2 mutant p.Leu1007fsX1008 (rs2066847) was highly associated with penetrating CD (p = 0.01), the development of fistulas (p = 0.01) and stenoses (p = 0.01), and ileal disease localization (p = 0.03). Importantly, the *NOD2* SNP rs2066847 was a strong separator between an aggressive and a mild course of CD (p = 2.99×10^−5^), while the *FOXO3A* SNP rs12212067 did not separate between mild and aggressive CD behavior in our cohort (p = 0.35). 96.2% of the homozygous NOD2 p.Leu1007fsX1008 carriers had an aggressive disease behavior compared to 69.3% of the patients with the *NOD2* wild-type genotype (p = 0.007).

**Conclusion/Significance:**

In clinical practice, the NOD2 variant p.Leu1007fsX1008 (rs2066847), in particular in homozygous form, is a much stronger marker for a severe clinical phenotype than the *FOXO3A* rs12212067 SNP for a mild disease course on an individual patient level despite its important impact on the inflammatory response of monocytes.

## Introduction

Crohn's disease (CD) and ulcerative colitis (UC) are the two main forms of inflammatory bowel disease (IBD) characterized by a chronic relapsing disease course based on a chronic inflammation of the intestine, frequently resulting in a stricturing and penetrating disease phenotype in CD patients. Since the introduction of biological therapies in the treatment of IBD, therapeutic options have dramatically improved [1]. Anti-TNF therapies have been proven in various clinical trials to alleviate the IBD disease course by rapidly inducing and maintaining clinical remission and mucosal healing in IBD patients [1]. These therapies are indispensable for the management of moderate to severe forms of IBD. Several novel compounds and “biosimilars” are currently undergoing clinical trials and will further increase the treatment options in IBD [1].

Therefore, it is necessary to personalize treatment algorithms according to the individual risk profile. A major task in daily clinical practice thus is the selection of the appropriate therapy for each CD patient [2]. For example, some CD patients will benefit from early initiation of biological therapies. The early prediction of the disease course after initial diagnosis therefore becomes increasingly important for subsequent therapeutic decisions. Several clinical, serological, and genetic factors have been investigated for disease prediction.

Likely the most relevant genetic factors associated with a complicated course of CD are the three common mutations of the NOD2 protein, p.Arg702Trp (rs2066844), p.Gly908Arg (rs2066847), and p.Leu1007fsX1008 (rs2066847), which have been associated with a complicated disease course of CD and in particular with a stricturing and penetrating disease behaviour. They have been described in multiply affected IBD families as a main genetic risk factor for CD susceptibility [3], [4], [5], [6], [7], [8], [9]. This phenotype is especially well-marked in the presence of the frameshift mutation p.Leu1007fsX1008 in exon 11 of the *NOD2* gene [3], [4], [5], [6], [7], [8], [10] which therefore has been successfully used as a genetic marker for therapeutic decisions in CD [4], [5].

Since the identification of *NOD2*, several genome-wide association studies (GWAS) were performed to identify new genetic loci for disease prediction in patients with IBD [11], [12], [13]. A recent meta-analysis identified and confirmed a total of 163 susceptibility loci for IBD [11], but only the *NOD2* mutation status has been translated from initial genotype-phenotype correlations to a clinically useful genetic predictor [11], [14].

Genetic differences in the IL-2 and IL-7 cytokine pathways were also strongly correlated with disease course prognosis in CD [15], [16], [17]. In particular, a noncoding SNP was identified in the *FOXO3A* gene which was associated with CD prognosis although it was not disease-associated [15]. *FOXO3A* encodes FOXO3, a member of the fork-head box O family of transcription factors that is involved in the regulation of diverse transcriptional programs including cell-cycle control, metabolism, regulatory T cell development, and apoptosis [18], [19], [20], [21], [22], [23], [24]. The minor G allele of the *FOXO3A* SNP rs122112067 was associated with a milder course of CD most likely via limitation of inflammatory responses in monocytes through a FOXO3-driven pathway [15].

The reliable prediction of a mild disease course of CD would represent an essential improvement in daily clinical practice by accelerating and ensuring therapeutic decisions in patients with IBD, especially with regard to long-term treatment. The initial observation of an association between the minor G allele of the *FOXO3A* SNP rs122112067 and a milder disease course in CD was replicated in cohorts of the British population, but not verified in IBD populations outside of Great Britain. Therefore, our study represents the first attempt to replicate the association of the *FOXO3A* SNP rs122112067 with a milder CD phenotype outside the UK. In addition, it remains elusive whether this *FOXO3A* SNP represents a stronger modifier of the CD course than *NOD2* gene mutants, which are currently the most important genetic markers for a severe CD phenotype.

Therefore, we first aimed to analyze a potential correlation of the *FOXO3A* SNP rs122112067 genotype status with a mild disease course in a German CD patient cohort. Our second aim was to compare this *FOXO3A* SNP with the most strongly CD-associated *NOD2* mutations regarding the most important disease outcome variables such as stenoses, fistulas, and need for surgery. This study was aimed to clarify the clinical value of the *FOXO3A* SNP rs122112067 for therapeutic decisions on an individual patient level.

## Patients and Methods

### Ethics Statement

This study was approved by the local ethics committee (Medical Faculty of the Ludwig-Maximilians-University Munich). Written, informed consent was obtained from all patients prior to study inclusion. Study protocols were based on the ethical principles for medical research involving human subjects of the Helsinki Declaration.

### Study population

A total of 550 patients with CD were enrolled in this study. All CD patients were recruited from the University Hospital Munich-Grosshadern and from the University Hospital Munich-Innenstadt. Patients with indeterminate colitis were excluded. Phenotypic parameters were collected blinded to the results of the genotype analyses [25] and included demographic and clinical data (behaviour and anatomic location of IBD, disease-related complications, surgical or immunosuppressive therapy). Two senior gastroenterologists analyzed the data which were recorded by patient chart analysis and a detailed questionnaire based on an interview at the time of enrolment. The diagnosis of CD was based on endoscopic, radiological, and histopathological parameters, adhering to established international guidelines [26]. CD patients were classified according to the Montreal classification [27] including age at diagnosis (A), location (L), and behaviour (B) of disease.

To distinguish between aggressive and mild disease, the clinical course of CD was defined as “aggressive” in CD patients with a stricturing (B2) and/or penetrating disease behaviour (B3) and/or CD-related surgery. CD patients with a “mild” disease course had a non-stricturing or non-penetrating phenotype and no CD-related surgery (B1 according to the Montreal Classification) [27].

### DNA extraction and NOD2 genotyping

Genomic DNA was isolated from peripheral blood leukocytes by standard procedures using the DNA blood mini kit from Qiagen (Hilden, Germany). The *FOXO3A* variant rs12212067 was genotyped by PCR and melting curve analysis on a LightCycler 480 Instrument (Roche Diagnostics, Mannheim, Germany) as previously described in detail [25], [28], [29]. The results of the melting curve analyses were confirmed by DNA sequence analysis of samples representing all three possible genotypes. Sequences of primers and FRET probes and primer annealing temperatures used for genotyping and for sequence analysis are given in the supplementary data section (tables S1 and S2). Amplification and sequence analysis of *NOD2* exons 4, 8, and 11 were performed as described previously [4], [28], [30].

### Statistical analyses

Each genetic marker was tested for Hardy-Weinberg equilibrium in the control population. Single-marker allelic tests were performed with Pearson's χ2 test. All tests were two-tailed, and p-values <0.05 were considered significant. Odds ratios were calculated for the minor allele at each SNP. For evaluation of phenotypic consequences, we conducted logistic regression analyses. Data and genotype-phenotype analyses were done by using the PLINK v1.07 software (http://pngu.mgh.harvard.edu/purcell/plink/ [31] and R-2.4.1. (http://cran.r-project.org).

## Results

### Phenotypic characteristics of the study cohort

Table 1 shows the characteristics of the study population. A total of 550 CD patients were included in the analysis. There was an equal gender distribution (48.5% males), and the mean age was 41.5 years with a mean disease duration of almost 15 months. The majority of CD patients (72.5%) was between 17 and 40 years of age at first diagnosis of CD, and 14.4% of CD patients were younger than 17 years at the time of CD onset. Nearly three quarters of the patients had either stricturing or penetrating disease behaviour (25.0% stricturing disease behaviour (B2); 49.5% penetrating disease behaviour (B3)) and more than half of the patients (58.6%) required CD-related surgery (e.g., ileocecal resection, fistulectomy, colectomy, or ileostomy). Over 80% of CD patients were treated with immunosuppressive or biological therapies (table 1). Interestingly, almost one fifth of the patients (19.2%) had a positive family history of IBD, demonstrating a strong role of genetic factors in this patient population.

**Table 1:** Demographic characteristics of the CD study population based on the Montreal classification [27].

	Crohn's disease
	(n = 550)
**Gender**	
Male (%)	48.5
Female (%)	51.5
**Age** (yrs)	
Mean ± SD	41.5±13.8
Range	(10–80)
**Disease duration** (yrs)	
Mean ± SD	14.7±9.3
Range	(0–46)
**Body mass index** (kg/m^2^)	
Mean ± SD	23.1±4.1
Range	(13.1–40.8)
**Age at diagnosis** (n = 535)	
≤16 years (A1)	77 (14.4%)
17–40 years (A2)	388 (72.5%)
>40 years (A3)	70 (13.1%)
**Location** (n = 535)	
Terminal ileum (L1)	79 (14.8%)
Colon (L2)	65 (12.1%)
Ileocolon (L3)	385 (72.0%)
Upper GI (L4)	6 (1.1%)
Any ileal involvement (L1+L3)	464 (86.7%)
**Behaviour** 1 (n = 521)	
Non-stricturing, non-penetrat. (B1)	133 (25.5%)
Stricturing (B2)	130 (25.0%)
Penetrating (B3)	258 (49.5%)
**Clinical disease course** 2 (n = 550)	
**aggressive**	403 (73.3%)
**mild**	147 (26.7%)
**Use of immunosuppressive agents** 3 (n = 530)	425 (80.2%)
	
**Surgery because of CD** 4 (n = 507)	297 (58.6%)
**Fistulas** (n = 516)	258 (50%)
**Perianal fistulas** (n = 516)	58 (11.2%)
**Stenoses** (n = 518)	329 (63.5%)
**Positive family history of IBD** (n = 385)	74 (19.2%)

For each variable, the number of patients included is given.

1 Disease behaviour was defined according to the Montreal classification [27]. The stricturing disease phenotype was defined as the presence of stenoses without penetrating disease. The diagnosis of stenoses was made surgically, endoscopically, or radiologically (using MR enteroclysis).

2 The clinical course of CD was furthermore defined as “aggressive” in CD patients with a stricturing and/or penetrating disease behaviour and/or when CD-related surgery became necessary.

3 Immunosuppressive agents included azathioprine, 6-mercaptopurine, methotrexate, infliximab, and/or adalimumab.

4 Only surgery related to CD-specific problems (e.g., ileocecal resection, fistulectomy, colectomy, and ileostomy) was included.

### Impact of the *FOXO3A* variant rs12212067 on the phenotype of Crohn's disease

The table S3 gives an overview over the observed minor allele frequencies of the *FOXO3A* SNP rs12212067 (T>G) and the frequencies of the three main NOD2 mutants p.Arg702Trp (rs2066844), p.Gly908Arg (rs2066845), and p.Leu1007fsX1008 (rs2066847). Interestingly, the minor G allele frequency of the *FOXO3A* SNP rs12212067 was with 12.7% identical to the allele frequency of the frameshift mutation p.Leu1007fsX1008 in exon 11 of the *NOD2* gene.

To evaluate the phenotypic consequences of the *FOXO3A* variant rs12212067 in CD patients, we performed a detailed genotype-phenotype correlation of the whole cohort of 550 CD patients including carriers of the three main *NOD2* mutants (table 2). Four hundred and fourteen patients were homozygous carriers of the major T allele (75.3%, *FOXO3A* WT), 132 patients (24.0%) were heterozygous carriers of the minor G allele, and 4 CD patients (0.7%) were homozygous for the G allele in *FOXO3A* (table 2). No significant differences were observed between the wild-type and minor allele carriers of the *FOXO3A* variant rs12212067 with respect to the age at diagnosis, disease duration, disease behaviour, disease location, need for CD-related surgery, and use of immunosuppressives (table 2).

**Table 2:** Association between the *FOXO3A* rs12212067 genotype and CD disease characteristics based on the Montreal classification [27].

*FOXO3A*	(1)	(2)	(1) vs. (2)	(1) vs. (2)	(1) vs. (2)
rs12212067	TT	TG/GG	p-value	OR	95% CI
genotype status	n = 414	n = 136			
**Male sex** (n = 550)	192 (46.4%)	75 (55.1%)	0.076	0.70	0.48–1.04
**Age at diagnosis** (yrs, n = 535, based on median OR+CI for > median)					
Mean ± SD	27.7±12.3	25.5±10.0	0.437	1.17	0.79–1.73
Range	(2–70)	(4–57)			
**Disease duration** (yrs, n = 502, based on median OR+CI for > median)					
Mean ± SD	14.5±9.2	15.4±9.5	0.235	0.78	0.52–1.17
Range	(0–46)	(0–40)			
**Body mass index** (n = 442, based on median OR+CI for > median)					
Mean ± SD	23.2±4.0	22.7±4.1	0.223	1.31	0.85–2.03
Range	(13.1–36.6)	(15.7–40.8)			
**Age at diagnosis**	(n = 403)	(n = 132)			
≤16 years (A1)	58 (14.4%)	19 (10%)	1	0.99	0.57–1.75
17–40 years (A2)	287 (71.2%)	101 (76%)	0.237	0.76	0.48–1.20
>40 years (A3)	58 (14.4%)	12 (14%)	0.120	1.68	0.87–3.24
**Location** (n = 535)					
	(n = 401)	(n = 134)			
Terminal ileum (L1)	58 (14.5%)	1 (15.7%)	0.733	0.91	0.53–1.57
Colon (L2)	48 (12.0%)	17 (12.7%)	0.826	0.94	0.52–1.70
Ileocolon (L3)	289 (72.0%)	96 (71.6%)	0.924	1.02	0.66–1.58
Upper GI (L4)	6 (1.5%)	0 (0%)	0.676	1.12	0.65–1.93
Any ileal involvement	347 (86.5%)	117 (87.3%)	0.818	0.93	0.52–1.67
(L1+L3)					
**Behaviour** 1 (n = 521)					
	(n = 391)	(n = 130)			
Non-stricturing, Non-penetrat. (B1)	93 (23.8%)	41 (31.5%)	0.252	0.78	0.50–1.12
Stricturing (B2)	100 (25.6%)	29 (22.3%)	0.346	1.24	0.79–1.96
Penetrating (B3)	198 (50.6%)	60 (46.2%)	0.856	1.04	0.70–1.55
**Use of immunosuppressive agents** 2 (n = 530)					
	(n = 396)	(n = 134)			
	317 (80.0%)	108 (80.6%)	0.890	0.97	0.59–1.58
**Surgery because of CD** 3 (n = 507)					
	(n = 380)	(n = 127)			
	221 (58.2%)	76 (59.8%)	0.739	0.93	0.62–1.40
**Fistulas** (n = 516)					
	(n = 387)	(n = 129)			
	198 (51.2%)	59 (45.7%)	0.286	1.24	0.83–1.85
**Perianal fistulas** (n = 513)					
	45/387 (11.6%)	13/129 (10.1%)	0.611	1.184	0.62–2.27
**Stenoses** (n = 518)					
	(n = 390)	(n = 128)			
	249 (63.9%)	77 (60.2%)	0.519	1.14	0.76–1.72

For each variable, the number of patients included is given.

1 Disease behaviour was defined according to the Montreal classification. A stricturing disease phenotype was defined as presence of a stenosis without penetrating disease. The diagnosis of stenoses was made surgically, endoscopically, or radiologically (using MR enteroclysis).

2 Immunosuppressive agents included azathioprine, 6-mercaptopurine, methotrexate, infliximab, and/or adalimumab.

3 Only surgery related to CD-specific problems (e.g., ileocecal resection, fistulectomy, colectomy, and ileostomy) was included.

Furthermore, no association with a mild disease course was found in the four CD patients being homozygous carriers of the *FOXO3A* minor allele. Three of these individuals carried none of the three *NOD2* mutations analyzed, while one CD patient was a heterozygous carrier of the NOD2 p.Arg702Trp (rs2066844) substitution. Two of these four patients had a stricturing or penetrating disease behaviour (none of them was carrier of one of the three main CD-associated NOD2 mutations analyzed); one of these patients needed CD-related surgery (table 2). Three of these patients had mainly ileocolonic disease; the female CD patient (also not carrying one of the three *NOD2* mutations) had isolated colonic disease. All patients were treated with immunosuppressive agents; three of them had anti-TNF therapy with infliximab.

### Analysis regarding an aggressive versus mild Crohn's disease course using the genotype status of the *FOXO3A* variant rs12212067 and the *NOD2* variants rs2066844, rs2066845, and rs2066847

To analyze the association between the *FOXO3A* SNP rs122112067 and the subsequent disease course in our cohort of 550 CD patients, we next defined the clinical course as “aggressive” in patients with a stricturing (B2) and/or a fistulising disease behaviour (B3) and/or when CD-related surgery (e.g., ileocecal resection, fistulectomy) became necessary. A “mild” clinical disease course was defined by inflammatory disease behaviour with no complications (B1) (e.g., no need for surgery). The majority of 403 CD patients had an aggressive disease course (73.3%), compared to 147 patients (26.7%, table 1) with only mild inflammatory disease.

The *FOXO3A* rs122112067 minor allele frequencies did not differ significantly between the two groups (table 3; allele frequency of 12.2% in the aggressive CD group vs. 14.3% in the group with mild CD (p = 0.35)). Similarly, for the *NOD2* variants rs2066844 and rs2066845 no significant differences were observed between the two groups (rs2066844: 9.7% vs. 9.9% aggressive vs. mild; p = 0.93) (rs2066845: 4.6% vs. 3.4%; p = 0.39) regarding the minor allele frequency (table 3).

**Table 3:** CD phenotype stratified by disease course.

		Aggressive disease behaviour	Mild disease behaviour	
Genetic marker		(stenosis and/or fistula and/or CD-related surgery; n = 403)	(no stenosis, no fistula, no CD-related surgery = inflammatory disease type; n = 147)	p-value
**rs12212067**	**G-allele (%)**	**98/806 (12.2)**	**42/294 (14.3)**	
				0.35
	T-allele (%)	708/806 (87.8)	252/294 (85.7)	
**rs2066844**	**T-allele (%)**	**78/806 (9.7)**	**29/294 (9.9)**	
				0.93
	C-allele (%)	728/806 (90.3)	265/294 (90.1)	
**rs2066845**	**C-allele (%)**	**37/806 (4.6)**	**10/294 (3.4)**	
				0.39
	G-allele (%)	769/806 (95.4)	284/294 (86.6)	
**rs2066847**	**X-allele (%)**	**123/806 (15.3)**	**17/294 (5.8)**	
				**2.99×10^−5^**
	C-allele (%)	683/806 (84.7)	277/294 (94.2)	

Aggressive disease behaviour was defined as stricturing and/or penetrating disease course ± CD-related surgery. Given are the allele frequencies (upper line: minor allele frequency in bold, bottom line: major allele frequency) for CD patients with an aggressive or with a mild disease behaviour, respectively, for the *FOXOA3* SNP rs12212067 and for the three main *NOD2* mutants rs2068844, rs2066845, and rs2066847. Only the p.Leu1007fsX1008 frameshift mutation in exon 11 of the *NOD2* gene (rs2066847) was significantly associated with an aggressive disease behaviour in patients with CD (p = 2.99×10^−5^, Chi-squared test). For the two other NOD2 mutants and especially for the *FOXO3A* SNP rs12212067, no significant differences were seen in the allele frequencies in patients with aggressive vs. mild disease behaviour. Some parts of the *NOD2* data have already been published elsewhere [4], [28], [30].

In contrast, the NOD2 frameshift mutation p.Leu1007fsX1008 (rs2066847) in exon 11 showed significant differences in the minor allele frequency in patients with aggressive disease compared to those with mild disease. In individuals with aggressive CD, the minor allele frequency of p.Leu1007fsX1008 was 15.3% versus 5.8% in patients with mild CD (p = 2.99×10^−5^; table 3).

### Genotype-phenotype analysis of *FOXO3A* and *NOD2* variants

Since the three NOD2 mutants p.Arg702Trp (rs2066844), p.Gly908Arg (rs2066845), and p.Leu1007fsX1008 (rs2066847) are the strongest genetic markers associated with the subsequent disease course in CD, we performed a genotype-phenotype correlation for the *FOXO3A* SNP rs12212067 in CD patients not carrying one of the three NOD2 mutations tested (table 4). Among the 550 CD patients, a total of 219 patients had the TT genotype of the *FOXO3A* SNP (*FOXO3A* wild-type (WT)). Seventy-three patients were rs12212067 minor G allele carriers (71 heterozygous and 2 homozygous; table 4). No significant differences were observed regarding the age at diagnosis, disease duration, location of the disease according to the Montreal classification, and disease behaviour in wild-type (WT) compared to the minor G allele carriers (table 4). In both subcohorts (TT/NOD2 WT group and TG/GG/NOD2 WT group), almost 80% of patients were treated with immunosuppressive agents or biological therapies (79% in the TT/NOD2 WT group vs. 78.3% in the TG/GG/NOD2 WT group). Almost every second CD patient developed fistulas (48.0% vs. 46.1%), and 62.1% in the TT/NOD2 WT group developed stenoses compared to 56.3% in the TG/GG/NOD2 WT group. More than half of all CD patient needed CD-related surgery (55.0% vs. 56.9%) (table 4).

**Table 4:** CD phenotype stratified by the *FOXO3A* rs12212067 genotype in CD patients carrying none of the three NOD2 mutations analyzed (NOD2 wild-type).

*FOXO3A* rs12212067	(1)	(2)	(1) vs. (2)	(1) vs. (2)	(1) vs. (2)
genotype status	TT/NOD2	GG/TG/NOD2	p value	OR	95% CI
	wild-type	wild-type			
	n = 219	n = 71			
**Male sex** (n = 290)	94 (43%)	36 (50.7%)	0.253	0.73	0.43–1.25
**Age at diagnosis** (yrs, n = 280, based on median OR+CI for > median)					
Mean ± SD	28.8 ± 12.1	26.4 ± 10.8	0.374	1.28	0.74–2.22
Range	(6–70)	(4–57)			
**Disease duration** (yrs, n = 262, based on median OR+CI for > median)					
Mean ± SD	14.5±9.4	16.4±9.4			
Range	(0–42)	(1–40)	0.148	1.53	0.86–2.71
**Body mass index** (n = 223, based on median OR+CI for > median)					
Mean ± SD	23.3±4.0	23.2±3.8	0.962	0.99	0.53–1.82
Range	(15.6–33.6)	(16.5–31.9)			
**Age at diagnosis**	(n = 213)	(n = 67)			
≤16 years (A1)	25 (11.7%)	8 (11.9%)	0.964	0.98	0.42–2.29
17–40 years (A2)	155 (72.8%)	52 (77.6%)	0.432	0.77	0.40–1.47
>40 years (A3)	33 (15.5%)	7 (10.5%)	0.307	1.57	0.66–3.74
**Location** (n = 280)					
	(n = 211)	(n = 69)			
Terminal ileum (L1)	33 (15.6%)	6 (8.7%)	0.154	1.95	0.78–4.87
Colon (L2)	28 (13.3%)	12 (17.4%)	0.397	0.73	0.35–1.52
Ileocolon (L3)	145 (68.7%)	51 (73.9%)	0.415	0.78	0.42–1.43
Upper GI (L4)	5 (2.4%)	0 (0%)	0.502	1.30	0.61–2.76
Any ileal involvement	178 (84.4%)	57 (82.6%)	0.731	1.14	0.55–2.34
(L1+L3)					
**Behaviour** 1 (n = 269)					
	(n = 204)	(n = 65)			
Non-stricturing, Non-penetrat. (B1)	55 (27%)	21 (32.3%)	0.550	0.83	0.46–1.52
Stricturing (B2)	52 (25.5%)	14 (21.5%)	0.289	1.42	0.74–2.72
Penetrating (B3)	97 (47.5%)	30 (46.2%)	0.675	0.89	0.51–1.56
**Use of immunosuppressive agents** 2 (n = 278)					
	(n = 209)	(n = 69)			
	167 (79%)	54 (78.3%)	0.77	1.10	0.57–2.15
**Surgery because of CD** 3 (n = 261)					
	(n = 196)	(n = 65)			
	108 (55%)	37 (56.9%)	0.266	0.93	0.53–1.64
**Fistulas** (n = 266)					
	(n = 201)	(n = 65)			
	97 (48%)	30 (46.1%)	0.768	1.09	0.62–1.91
**Perianal fistulas** (n = 266)					
	23/201 (11.4%)	6/65 (9.2%)	0.603	1.29	0.50–3.31
**Stenoses** (n = 268)					
	(n = 204)	(n = 64)			
	126 (62.1%)	36 (56.3%)	0.432	1.26	0.71–2.22

Group (1): CD patients carrying the rs12212067 T variant in homozygous form (219 patients with the TT genotype and no further NOD2 mutant), group (2): CD patients with the *FOXO3A* rs12212067 GG or TG genotype and no further NOD2 mutation.

1 According to the Montreal classification, a stricturing disease phenotype was defined as the presence of a stenosis without penetrating disease. The diagnosis of stenoses was made surgically, endoscopically, or radiologically (using MR enteroclysis).

2 Immunosuppressive agents included azathioprine, 6-mercaptopurine, methotrexate, infliximab, and/or adalimumab.

3 Only surgery related to CD-specific problems (e.g., ileocecal resection, fistulectomy, colectomy, and ileostomy) was included.

In summary, no significant phenotypic differences were identified between the different *FOXO3A* genotypes. There were also no significant associations when both *FOXO3A* and *NOD2* SNPs were analysed in combination. In our patient cohort, the *FOXO3A* G allele was not associated with a milder CD course.

### Homozygosity for the NOD2 frameshift mutation p.Leu1007fsX1008 (rs2066847) is strongly associated with complicated CD

Twenty-six of the 550 CD patients (4.7%) were homozygous for the frameshift mutation p.Leu1007fsX1008 (rs2066847) in exon 11 of the *NOD2* gene (table S4). These patients were nominally younger when diagnosed with CD as compared with those carrying none of the three tested NOD2 mutations (NOD2 wild-type (WT); mean age of 22.4 years at diagnosis versus 28.2 years in NOD2 WT patients (23.1%), p = 0.09) (Table S4). The main disease location was the terminal ileum in 23.1% (n = 6) of these 26 patients, compared to 32 out of 280 (11.4%) NOD2 WT patients with a L1 disease localization (p = 0.027). Twenty-three of the twenty-six CD patients with the homozygous p.Leu1007fs genotype (88.5%) suffered from intestinal stenoses vs. 162/271 (59.8%) of NOD2 WT patients (p = 0.01), while 19/26 (73.1%) had fistulas vs. 127/269 (47.2%) of NOD2 WT patients (p = 0.01). A total of 65.4% (17/26) patients with the homozygous p.Leu1007fs genotype needed CD-related surgery compared to 54.5% (145/266) of the NOD2 WT patients (p = 0.34) (Table S4).

In summary, 25 of the 26 CD patients carrying the p.Leu1007fsX1008 mutation in homozygous form (96.2%) had an aggressive disease behavior compared to 69.3% (201/290) of the patients with the *NOD2* wild-type genotype (p = 0.007).

### The presence of the minor G allele of *FOXO3A* rs12212067 does not protect against a severe disease phenotype in homozygous carriers of the NOD2 p.Leu1007fs mutation

An additional sub-analysis showed that six of the 26 patients with the *NOD2* frameshift mutation on both alleles were also heterozygous carriers of the minor G allele of the *FOXO3A* SNP rs12212067. Based on our definitions for “mild” and “aggressive” disease, five of these patients had an aggressive disease phenotype (83.3%), while one CD patient had mild disease. This patient was diagnosed with CD at the age of 13 years and needed immunosuppressive treatment early on. This demonstrates, that in carriers of the minor G allele of the *FOXO3A* SNP rs12212067, no clinically protective effect on the disease course is observed if the patients are homozygous carriers of the 1007fs NOD2 variant, further reinforcing our conclusion that the *FOXO3A* rs12212067 SNP minor G allele is not associated with a mild(er) disease course in our patient cohort. In summary, there is clinically a much stronger effect of NOD2 1007fs homozygosity than carrier status of the *FOXOA3* rs12212067 variant, limiting the usefulness of this *FOXO3A* variant in clinical practice for therapeutic decisions for individual patients, despite its effect on the inflammatory response of monocytes through the FOXO3-driven pathway [15].

## Discussion

With the evolution of new medical therapies for the treatment of IBD, the evaluation of an individualized treatment approach for each IBD patient becomes more important. For defining the most appropriate treatment algorithm in CD patients, prediction of the subsequent disease course is indispensable for lowering the risk of overtreatment and limiting the use of potentially harmful immunosuppressive therapy in mild CD, and for initiating a therapy with biologicals in early “aggressive” CD to lower CD-related complications and surgery.

The present study demonstrates that the NOD2 mutant p.Leu1007fsX1008 (rs2066847) strongly correlates with a severe CD course. The association between the *FOXO3A* SNP rs12212067 and a mild (or aggressive) disease behavior, in contrast, was not significant in our patient cohort. However, a post-hoc power calculation demonstrated that a very large sample size of 4140 CD patients would be necessary to demonstrate (with 80% power) statistical significance in our study cohort if the effect size (OR 0.83) and the allele frequency for the G allele for the *FOXO3A* SNP rs12212067 (0.127) would remain stable in our study. However, such a large cohort can only be collected in a large multicenter study. This study is therefore not sufficient to reject the hypothesis of an association of the FOXO3A SNP rs12212067 with a mild disease phenotype; however, as demonstrated by the very robust association of the *NOD2* 1007fs variant with an aggressive disease phenotype (p = 2.99×10^−5^), the cohort size is sufficient to detect clinically relevant associations. Considering the aim of our study, which tried to clarify the clinical value of the *FOXO3A* SNP rs122112067 for therapeutic decisions on an individual patient level, our data strongly suggest that - in contrast to the strong effect of *NOD2* 1007fs homozygosity – the *FOXO3A* SNP rs122112067 is not helpful for individual therapeutic decision given its very low predictive value for certain CD phenotypes. However, combining our cohort with the British CD cohort, the association of *FOXO3A* SNP rs12212067 with a mild CD phenotype remains significant (p = 1.58×10^−8^).

Our data confirmed that the *NOD2* genotype status is currently the strongest genetic marker associated with a severe CD course. This observation is supported by several previous reports that demonstrated the *NOD2* genotype as a marker for complicated CD [2], [4], [6], [8], [11], [28], [30].

An additional explanation for the observed differences between the German and the British cohort may be different definitions of “aggressive” and of “indolent”/“mild” CD. We used a stricter definition of “aggressive” CD, as we only included CD patients with penetrating and/or stricturing CD, with and without CD-related surgery. Furthermore, over 80% of our patients needed immunosuppressive therapy. Seventy-three percent of these patients therefore had an aggressive disease compared to 63% of patients in the cohort of Lee et al. [15]. Unfortunately, there is no information on the *NOD2* genotype status in the study of Lee et al. Therefore, it remains unclear if the results of the British study can be explained independently of the *NOD2* genotype.

As mentioned above, one limitation of our study is the lower number of CD patients included in the overall analysis (550 CD patients in our study vs. a total of 2.985 CD patients in the study of Lee et al. [15]). However, our study cohort is phenotypically characterized in more detail than the cohorts of the British study, providing disease phenotypes and clinical information for all patients. Nevertheless, the *FOXO3A* minor allele frequencies (see above) regarding the proportions of CD patients with “mild” disease behaviour were comparable between the British and our German IBD cohort (13.1% in patients with “indolent” CD in the British IBD cohort versus 14.3% in patients with “mild” CD in our IBD cohort).

Although the follow-up period in our patient group with mild disease (45.6 months) was comparable to that of patients with severe disease (49.8 months), it cannot be excluded that patients with mild CD convert later to a severe disease phenotype which may take in some patients up to 20 years. However, given that the definition of “mild disease” in the study by Lee et al. [15] was defined as “disease of greater than 4 years duration, for which immunomodulators or intestinal resections had never been required” and our median follow-up in both CD subgroups was approximately four years, we assume that major differences in disease duration are not the main reason for the observed differences in the German and the British study.

In conclusion, in clinical practice, the *FOXO3A* SNP rs12212067 is – in contrast to the 1007fs NOD2 variant not a relevant genetic marker for therapeutic decisions on an individual patient level despite its impact on the inflammatory response of monocytes [15]. Our study indicates that the clinical value of the three NOD2 mutants for relevant therapeutic decisions is mainly limited to the frameshift mutation p.Leu1007fs in exon 11. Especially CD patients carrying this alteration in homozygous form have a complicated disease course. In these patients, a “top down” approach with an early initiation of anti-TNF therapy and/or immunosuppressive therapy seems to be justified.

## Supporting Information

Table S1 **Sequences of primers and FRET probes used for the genotyping the** ***FOXO3A*** **variant rs12212067.**
(DOC)

Table S2 **Primers used for amplification and sequencing of the** ***NOD2*** **exons 4, 8, and 11.**
(DOC)

Table S3 **Shown are the minor allele frequencies (MAF) of the** ***FOXO3A*** **SNP rs12212067 and the three main Crohn's disease-associated** ***NOD2*** **variants rs2066844 (p.Arg702Trp), rs2066845 (p.Gly908Arg), and rs2066847 (p.Leu1007fsX1008) in patients with Crohn's disease.**
(DOC)

Table S4 **Association between the** ***NOD2*** **rs2066847 genotype and Crohn's disease characteristics based on the Montreal classification [27].**
(DOC)
