# Supplementary material for: The NOD2 p.Leu1007fsX1008 Mutation (rs2066847) Is a Stronger Predictor of the Clinical Course of Crohn's Disease than the FOXO3A Intron Variant rs12212067
Source: PLoS One. 2014 Nov 3;9(11):e108503. doi: 10.1371/journal.pone.0108503 (PMC4217717; doi:10.1371/journal.pone.0108503)
Supplement: Table S1 — Sequences of primers and FRET probes used for the genotyping the FOXO3A variant rs12212067. (DOC) [file pone.0108503.s001.doc]

| **Primers used for *FOXO3* rs12212067 genotyping** | |
| --- | --- |
| **sense** | **anti-sense** |
| 5′- CTATACGCATACGTTGTTGGAGGT -3′ | 5′- AGCAGGCAAGTACAGGTGGAT -3′ |
| FRET simple probe | |
| AGT**A**TTCAGCTATCCCCCCTTAAAAAGCAT | |

**Supplemental table S1.** Sequences of primers and FRET probes used for the genotyping the *FOXO3A* variant rs12212067.
