# Supplementary material for: The NOD2 p.Leu1007fsX1008 Mutation (rs2066847) Is a Stronger Predictor of the Clinical Course of Crohn's Disease than the FOXO3A Intron Variant rs12212067
Source: PLoS One. 2014 Nov 3;9(11):e108503. doi: 10.1371/journal.pone.0108503 (PMC4217717; doi:10.1371/journal.pone.0108503)
Supplement: Table S2 — Primers used for amplification and sequencing of the NOD2 exons 4, 8, and 11. (DOC) [file pone.0108503.s002.doc]

| **Exon** | **Primers used for *NOD2* genotyping** | | **length of product (bp)** |
| --- | --- | --- | --- |
|  | **sense** | **anti-sense** |  |
| 4 | 5′- TTAGGTCCCGTCTTCACCATG -3′ | 5′- CTCCCACACTTAGCCTTGATGG -3′ | 1936 |
| 8 | 5′- GGAGGAGGACTGTTAGTTCATGTCTAG -3′ | 5′- CTCCTCCCTCTTCACCTGATCTC -3′ | 223 |
| 11 | 5′- GACAGGTGGGCTTCAGTAGACTG –3′ | 5′- GATCCTCAAAATTCTGCCATTCC -3′ | 293 |

| **Exon** | **Primers used for *NOD2* sequencing** | |  |
| --- | --- | --- | --- |
| 4 | 5′- CTCCCACACTTAGCCTTGATGG -3′ | Anti-sense | |
| 8 | 5′- GGAGGAGGACTGTTAGTTCATGTCTAG -3′ | sense | |
| 11 | 5′- GACAGGTGGGCTTCAGTAGACTG –3′ | sense | |

**Supplemental table S2.** Primers used for amplification and sequencing of the *NOD2* exons 4, 8, and 11.
