# Supplementary material for: The NOD2 p.Leu1007fsX1008 Mutation (rs2066847) Is a Stronger Predictor of the Clinical Course of Crohn's Disease than the FOXO3A Intron Variant rs12212067
Source: PLoS One. 2014 Nov 3;9(11):e108503. doi: 10.1371/journal.pone.0108503 (PMC4217717; doi:10.1371/journal.pone.0108503)
Supplement: Table S3 — Shown are the minor allele frequencies (MAF) of the FOXO3A SNP rs12212067 and the three main Crohn's disease-associated NOD2 variants rs2066844 (p.Arg702Trp), rs2066845 (p.Gly908Arg), and rs2066847 (p.Leu1007fsX1008) in patients with Crohn's disease. (DOC) [file pone.0108503.s003.doc]

| **Gene marker** | **Minor allele** | **Crohn’s disease** (n=550) |
| --- | --- | --- |
|  |  | **MAF (%)** |
| rs12212067 | G | 12.7 |
| rs2066844 p.Arg702Trp | T | 9.7 |
| rs2066845 p.Gly908Arg | C | 4.3 |
| rs2066847 p.Leu1007fsX1008 | insC | 12.7 |

**Supplemental Table S3.** Shown are the minor allele frequencies (MAF) of the *FOXO3A* SNP rs12212067 and the three main Crohn’s disease-associated *NOD2* variants rs2066844 (p.Arg702Trp), rs2066845 (p.Gly908Arg), and rs2066847 (p.Leu1007fsX1008) in patients with Crohn’s disease.
