# Supplementary material for: The NOD2 p.Leu1007fsX1008 Mutation (rs2066847) Is a Stronger Predictor of the Clinical Course of Crohn's Disease than the FOXO3A Intron Variant rs12212067
Source: PLoS One. 2014 Nov 3;9(11):e108503. doi: 10.1371/journal.pone.0108503 (PMC4217717; doi:10.1371/journal.pone.0108503)
Supplement: Table S4 — Association between the NOD2 rs2066847 genotype and Crohn's disease characteristics based on the Montreal classification [27]. (DOC) [file pone.0108503.s004.doc]

| ***NOD2* rs2066847** | **(1)** | | **(2)** | **(1) vs. (2)** | **(1) vs. (2)** | **(1) vs. (2)** |
| --- | --- | --- | --- | --- | --- | --- |
| **genotype status** | **XX** | | **NOD2 WT** | **p-value** | **OR** | **95% CI** |
|  | n=26 | | n=290 |  |  |  |
| **Male sex** (n=316, of these 144 males) | | | | | | |
|  | 14 (46.4%) | | 130 (55.1%) | 0.38 | 1.44 | 0.64-3.21 |
| **Age at diagnosis** (yrs, n=306, based on median OR+CI for > median) | | | | | | |
| Mean ± SD | 22.4 ± 9.2 | | 28.2 ± 11.8 | 0.09 | 0.48 | 0.21-1.11 |
| Range | (11-52) | | (4-70) |  |  |  |
| **Disease duration** (yrs, n=286, based on median OR+CI for > median) | | | | | | |
| Mean ± SD | 13.9 ± 7.9 | | 14.9 ± 9.4 | 0.479 | 0.74 | 0.32-1.72 |
| Range | (2-27) | | (0-42) |  |  |  |
| **Body mass index** (n=246, based on median OR+CI for > median) | | | | | | |
| Mean ± SD | 22.0 ± 3.0 | | 23.3 ± 3.9 | 0.733 | 0.86 | 0.36-2.03 |
| Range | (17.2-29.3) | | (15.6-33.6) |  |  |  |
| **Age at diagnosis** | | | | | | |
|  | (n=26) | | (n=280) |  |  |  |
| ≤16 years (A1) | 6 (23.1%) | | 33 (11.8%) | 0.106 | 2.25 | 0.84-5.99 |
| 17-40 years (A2) | 19 (73.1%) | | 207 (73.9%) | 0.925 | 0.96 | 0.39-2.37 |
| > 40 years (A3) | 1 (3.8%) | | 40 (14.3%) | 0.167 | 0.24 | 0.03-1.82 |
| **Location** (n=306) | | | | | | |
|  | | (n=26) | (n=280) |  |  |  |
| Terminal ileum (L1) | | 6 (23.1%) | 32 (11.4%) | **0.03** | 2.75 | 1.12-6.75 |
| Colon (L2) | | 1 (3.8%) | 39 (13.9%) | 0.167 | 0.24 | 0.032-1.82 |
| Ileocolon (L3) | | 15 (57.7%) | 161 (57.5%) | 0.625 | 0.81 | 0.35-1.89 |
| Upper GI (L4) | | 4 (15.4%) | 48 (17.2 %) | 0.82 | 0.88 | 0.29-2.67 |
| Any ileal involvement | | 21 (80.8%) | 117 (68.9%) | 0.129 | 4.79 | 0.63-36.21 |
| (L1+L3) | |
| **Behaviour** ^1^ (n=295) | | | | | | |
|  | | (n=26) | (n=269) |  |  |  |
| Non-stricturing, non-penetrat. (B1) | | 1 (3.8%) | 76 (28.3%) | **0.032** | 0.20 | 0.05-0.87 |
| Stricturing (B2) | | 6 (23.1%) | 66 (24.5%) | 0.58 | 0.76 | 0.29-1.97 |
| Penetrating (B3) | | **19 (73.1%)** | 127 (47.2%) | **0.01** | 3.06 | 1.29-7.28 |
| **Use of immunosuppressive agents** ^2^ (n=304) | | | | | | |
|  | | (n=26) | (n=278) |  |  |  |
|  | | 21 (80.8%) | 221 (78.4%) | 0.878 | 1.08 | 0.39-3.00 |
| **Surgery because of CD** ^3^(n=287) | | | | | | |
|  | | (n=26) | (n=261) |  |  |  |
|  | | 17 (65.4%) | 145 (54.5%) | 0.338 | 1.51 | 0.65-3.51 |
| **Fistulas** (n=291) | | | | | | |
|  | | (n=26) | (n=269) |  |  |  |
|  | | 19 (73.1%) | 127 (47.2%) | **0.010** | 3.47 | 1.34-8.95 |
| **Perianal fistulas** (n=289) | | | | | | |
|  | | 2/26 (7.7%) | 29/269 (10.8%) | 0.646 | 0.70 | 0.16-3.14 |
| **Stenoses** (n=294) | | | | | | |
|  | | (n=26) | (n=271) |  |  |  |
|  | | 23 (88.5%) | 162 (59.8%) | **0.010** | 5.01 | 1.47-17.12 |

**Supplemental Table S4.** Association between the *NOD2* rs2066847 genotype and Crohn’s disease characteristics based on the Montreal classification . For each variable, the number of patients included is given.^1^ According to the Montreal classification, a stricturing disease phenotype was defined as presence of a stenosis without penetrating disease. The diagnosis of stenoses was made surgically, endoscopically, or radiologically (using MR enteroclysis). ^2^ Immunosuppressive agents included azathioprine, 6-mercaptopurine, methotrexate, infliximab, and/or adalimumab. ^3^ Only surgery related to CD-specific problems (e.g., ileocecal resection, fistulectomy, colectomy, ileostomy) was included.
